# A Novel CRISPR/Cas9 Screening Potential Index for Prognostic and Immunological Prediction in Low-Grade Glioma

**DOI:** 10.3389/fgene.2022.839884

**Published:** 2022-04-25

**Authors:** Xiangpan Li, Kewei Xiong, Dong Bi, Chen Zhao

**Affiliations:** ^1^ Department of Oncology, Renmin Hospital of Wuhan University, Wuhan, China; ^2^ School of Mathematics and Statistics, Central China Normal University, Wuhan, China

**Keywords:** CRISPR/Cas9, CNS—central nervous system, tumor microenvironment, machine learning, low-grade glioma

## Abstract

Glioma is a malignancy with the highest mortality in central nervous system disorders. Here, we implemented the computational tools based on CRISPR/Cas9 to predict the clinical outcomes and biological characteristics of low-grade glioma (LGG). The transcriptional expression profiles and clinical phenotypes of LGG patients were retrieved from The Cancer Genome Atlas and Chinese Glioma Genome Atlas. The CERES algorithm was used to screen for LGG-lethal genes. Cox regression and random survival forest were adopted for survival-related gene selection. Nonnegative matrix factorization distinguished patients into different clusters. Single-sample gene set enrichment analysis was employed to create a novel CRISPR/Cas9 screening potential index (CCSPI), and patients were stratified into low- and high-CCSPI groups. Survival analysis, area under the curve values (AUCs), nomogram, and tumor microenvironment exploration were included for the model validation. A total of 20 essential genes in LGG were used to classify patients into two clusters and construct the CCSPI system. High-CCSPI patients were associated with a worse prognosis of both training and validation set (*p* < 0.0001) and higher immune fractions than low-CCSPI individuals. The CCSPI system had a promising performance with 1-, 3-, and 5-year AUCs of 0.816, 0.779, 0.724, respectively, and the C-index of the nomogram model reached 0.743 (95% CI = 0.725–0.760). Immune-infiltrating cells and immune checkpoints such as PD-1/PD-L1 and POLD3 were positively associated with CCSPI. In conclusion, the CCSPI had prognostic value in LGG, and the model will deepen our cognition of the interaction between the CNS and immune system in different LGG subtypes.

## Introduction

Glioma remains the most aggressive malignancy of primary central nervous system (CNS) tumors globally (Cai et al., 2020). Patients suffering from glioma exhibit poor prognosis with a median survival of 1 year, and approximately only 5% of individuals survive more than 5 years (Ostrom et al., 2019). Low-grade glioma (LGG, grade II and III of the World Health Organization) is the most common primary malignancy in the brain and is mainly localized to the cerebral hemispheres (Bai et al., 2022). Although progress has been made in the treatment of glioma containing surgical resection, postoperative radiation, and chemotherapy, its high heterogeneity still leads to minimal therapeutic effectiveness (Zhang et al., 2022). Although multiple biomarkers for diagnosis and prognostic monitoring were investigated in previous studies and showed a promising outcome in clinical applications, still there is a limit to classification systems and reliable targets for treatment (Chen et al., 2021). Hence, it is urgent to seek for more advanced strategies for LGG management.

Clustered regularly interspaced short palindromic repeats (CRISPR) were first invented by using small guide RNA to target annotated genes interacting with clinical phenotypes, providing a maintainable modification for disorder treatment (Chen et al., 2019; Yu et al., 2022). Cas9 is an endonuclease that promotes the strand breaks of DNA. The target genes are expected to be altered when the proteins encoding Cas9 and the gene of small guide RNA are introduced into the cells. Because of using RNA molecules to mediate the binding, CRISPR-mediated genome editing has been recognized as a safe and long-term treatment for cancer (Rasul et al., 2022). The advanced technology allows us create cancer models with distinct mutations to identify early events, restore tumor suppressor gene functions, and correct harmful mutations (Rafii et al., 2022).

Recently, there are emerging studies using the CRISPR/Cas9 method in glioma, by which researchers have been able to identify several members responsible for glioma cell growth and proliferation such as USP8 and SOCS3 (MacLeod et al., 2019). Results from an *in vitro* and *in vivo* genome-wide CRISPR/Cas9 study suggested that the interplay of WEE1 and PKMYT1 had an important influence on the glioblastoma stem cell development (Toledo et al., 2015). Another clinic-oriented CRISPR/Cas9 library was conducted to investigate the drug resistance of glioma patients and revealed that E2F6 served as an underlying target for temozolomide treatment (Huang et al., 2019). However, the treatment efficacy of CRISPR/Cas9-based systems is actually influenced by the complex interactions among different gene types such as cell death-related genes, epigenetic genes, immune-related genes, viral oncogenes, and tumor microenvironment (TME) associated genes (Song et al., 2021). Substantial efforts have been made to integrate CRISPR/Cas9 into glioma studies, only a limited number of strategies in glioma biological and clinical exploration based on computational methods are executed.

In this study, we retrieved data from CRISPR/Cas9-based experiments to identify lethal genes of LGG in public datasets. A univariate Cox proportional hazard model and a random survival forest were employed to screen for prognosis-related genes. Subsequently, patients were classified into two subtypes by the unsupervised learning method. The survival outcomes and immune characteristics were investigated between the two clusters. Then we constructed a novel CRISPR/Cas9 screening index to simplify the clinical and TME features of LGG patients. In addition, the performance of the index was validated by independent prognostic analysis, receiver operating characteristic curves (ROCs), a combined diagnostic nomogram system, concordance index (C-index), and decision curve analysis (DCA).

## Materials and Methods

### Data Collection and Preprocessing

The transcriptional profile and clinical phenotypes of LGG patients were retrieved from The Cancer Genome Atlas (TCGA, https://portal.gdc.cancer.gov/) and the Chinese Glioma Genome Atlas (CGGA, http://www.cgga.org.cn/). The expression data of TCGA were processed into transcripts per million and transformed in log2 formation. The information of CGGA included “mRNAseq_693” and “mRNAseq_325” cohorts, and they were combined as a meta-cohort in the following statistical analysis. Then we performed a correction of batch effects in the “sva” package using the Combat algorithm. The cell lines and sample information collected from CNS based on CRISPR/Cas9 screening were downloaded from Depmap (https://depmap.org/portal/).

### Screening for Prognostic Candidate Genes in the Unsupervised Clustering

The dependency score of candidate genes in 10 glioma cell lines of CNS (Supplementary Table S1) was estimated by adopting the CERES algorithm (Meyers et al., 2017). Essential genes with CERES value less than -1 across 75% were selected to overlap the combined expression profile (Ho et al., 2021). A univariate Cox proportional hazard regression was first employed in preliminary screening with a threshold of *p* value < 0.001 in the TCGA cohort. Then a random survival forest was used to seek survival-related LGG-lethal genes with a threshold of 0.4. Nonnegative matrix factorization (NMF) with the brunet method was utilized to divide the glioma patients into different clusters.

### Exploration of the Survival and Tumor Microenvironment of Clustering Subtypes

We analyzed the differences in survival outcomes in clusters using the product-limit method and a log-rank test. The “estimation of stromal and immune cells in malignant tumors using expression data” (ESTIMATE) algorithm was run to quantify the tumor purity (Yoshihara et al., 2013). To assess the immune-infiltrating levels, MCPcounter (Becht et al., 2016) was conducted, and the Wilcoxon rank-sum test with a *p*-value < 0.05 was considered significant. Gene set variation analysis (GSVA) was performed to explore the enrichment pathways with “c2. cp.kegg.v7.4.symbols.gmt” and “c2.cp.reactome.v7.4.symbols.gmt” collected from the Molecular Signatures Database (MSigDB, http://www.gsea-msigdb.org/gsea/index.jsp). A value of *p* < 0.001 and the absolute value of log (Fold change/FC) > 0.1 were statistically significant for the enrichment analysis.

### Development of CRISPR/Cas9 Screening Potential Index System

The optimal survival-related glioma-lethal genes were assigned into a “risky component” with a hazard ratio (HR) > 1 and a “protective component” with HR < 1 in the univariate Cox model of the TCGA cohort. The CCSPI was modeled according to the enrichment score of “risky component” minus that of “protective component” calculated by a single sample GSEA (ssGSEA) (Barbie et al., 2009) utilizing the “GSVA” package. The patients were divided into low- and high-CCSPI groups based on the “survminer” package at the optimal cutoff of the training (TCGA) and validation (CGGA) sets, respectively. The survival differences between the two CCSPI subtypes were estimated by the log-rank test and Pearson chi-squared test. Meanwhile, the patients younger than 40 years old were stratified into a young group while those older than 40 were stratified into an old group. The ability to classify survival outcomes of CCSPI was validated in different clinicopathologic stratifications. The predictive efficacy and stratified ability were verified in the CGGA cohort.

### Validation of the CRISPR/Cas9 Screening Potential Index Model

Time-dependent ROC and AUC of 1, 3, and 5 years were conducted to assess the sensitivity and accuracy of the CCSPI system. The net benefits of CCSPI and other phenotypes were compared using decision curve analysis (DCA). A Pearson’s Chi-squared test was employed to investigate the distributions of clinicopathologic variables between the low- and high-CCSPI groups. An Independent prognostic exploration was employed to compare the prognostic value of clinicopathologic features and CCSPI by the Cox model, which was analyzed by “log minus log” curves for categorized data and Schoenfeld’s test for continuous data to verify the proportional hazard (PH) assumptions in advance. Nomograms based on age, histology, 1p/19q codeletion status and CCSPI, and calibration were used for combined diagnosis. C-index was calculated to evaluate the consistency of real and predicting survival outcomes. To further verify the performance of CCSPI, we compared different risk systems from other studies (Supplementary Table S2) measured by AUCs and C-index (Zhang et al., 2020; Luo et al., 2021; Zhao et al., 2021; Zhou et al., 2021; Wu et al., 2022; Zheng et al., 2022) in the CGGA validation cohort.

### Biological Functions and Immune Characteristics of the CRISPR/Cas9 Screening Potential Index Model

GSEA functional enrichment analysis in both low- and high-CCSPI groups was performed with the “c5.go.v7.4. symbols” gene set and “c2.cp.kegg.v7.4.symbols.gmt” gene set collected from MSigDB. Correlation approaches were used to analyze the associations of CCSPI, immune-infiltrating, and expression of immune checkpoints by Pearson coefficients.

## Results

### Identification of Lethal Genes and Clustering Subtypes Using Unsupervised Learning

**FIGURE 1:**
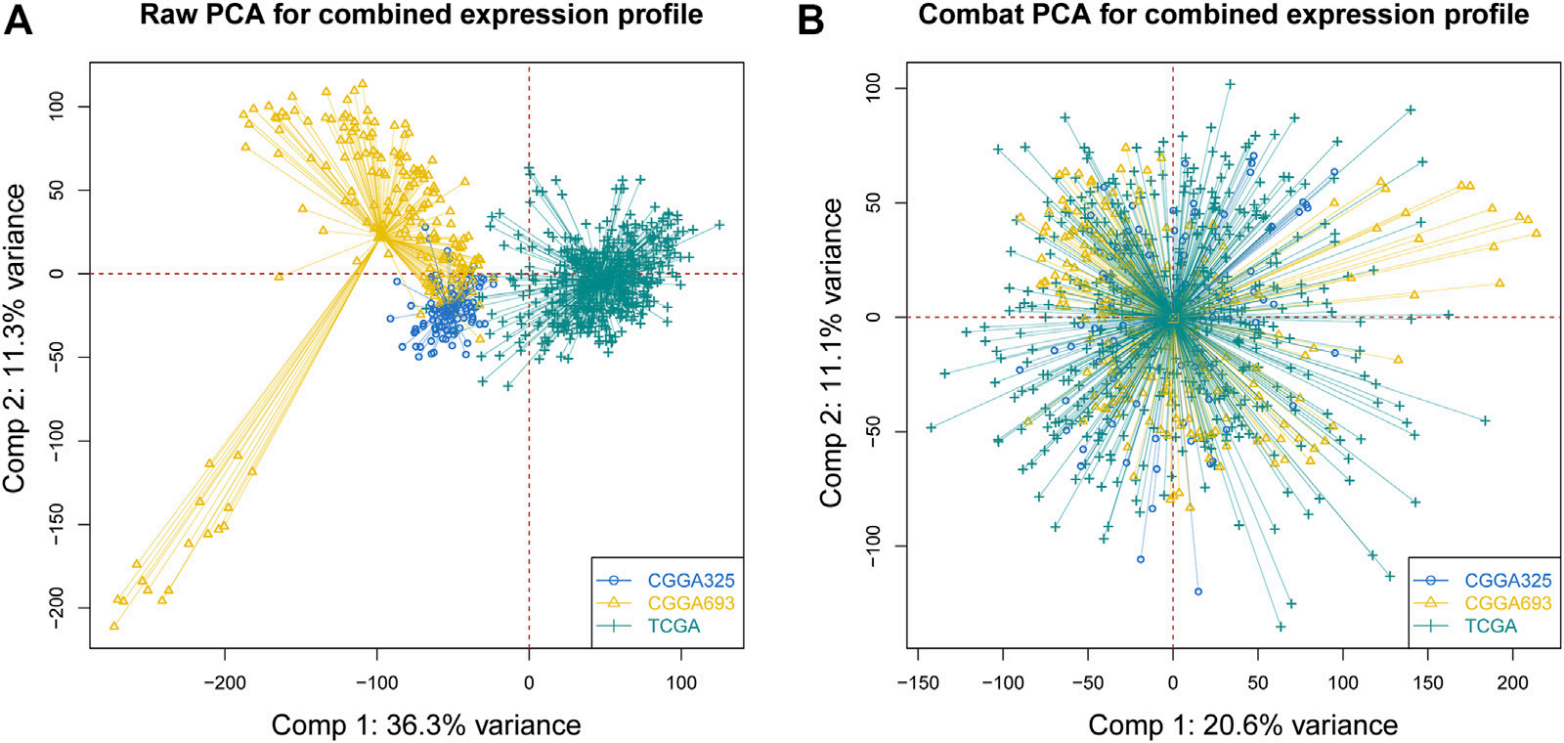
Correction of batch effects. **(A)** Raw PCA of three combined expression matrixes. **(B)** Combat PCA of three combined expression matrixes. (PCA: principal component analysis).

**FIGURE 2:**
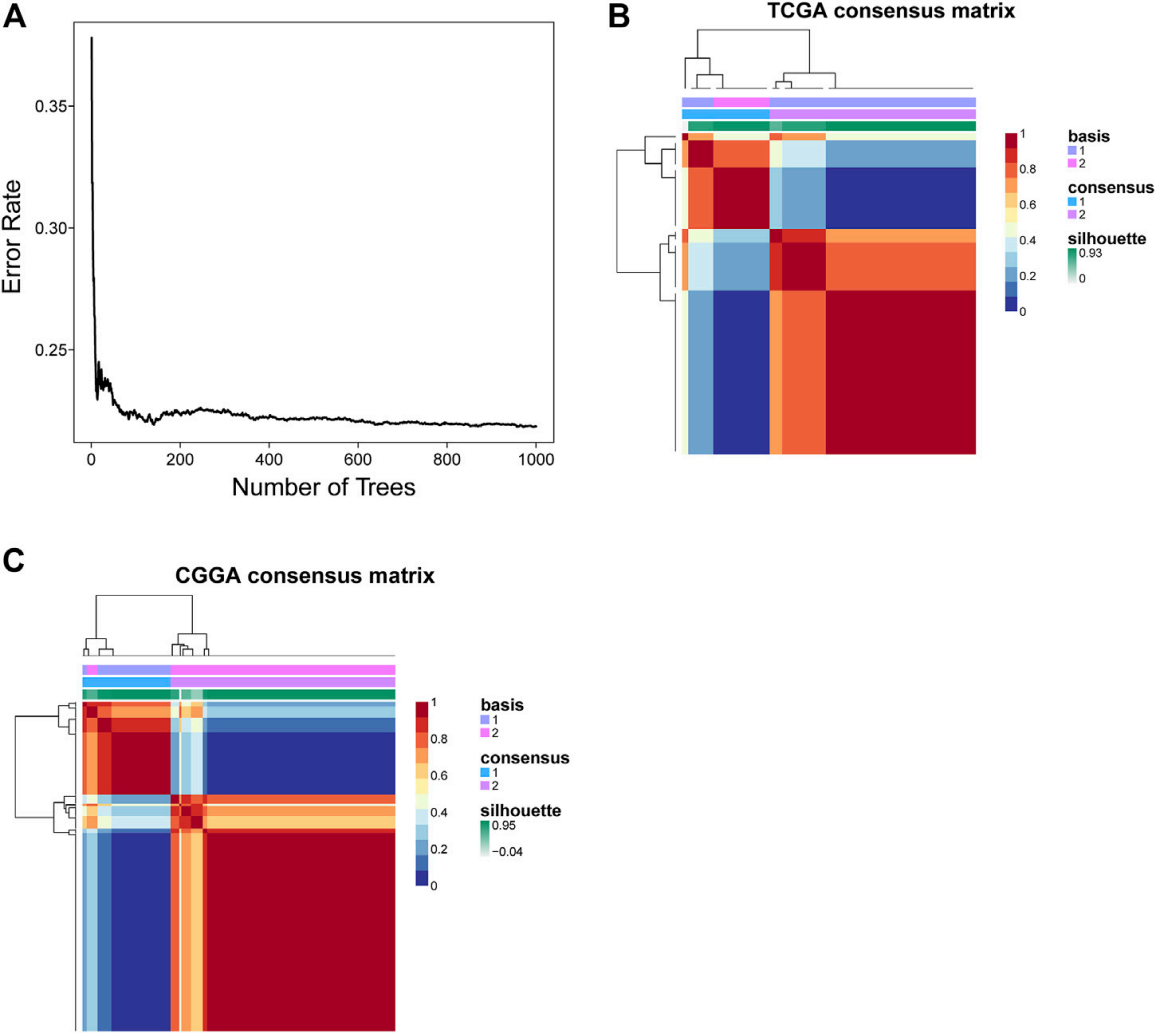
Identification of survival-related genes and clustering subtypes. **(A)** The relationship between the number of trees and error rate. **(B,C)** Consensus clustering heatmap of nonnegative matrix factorization of the TCGA and CGGA cohorts.

Before conducting the removal of batch effects, the first two components of the principal component principle (PCA) accounted for 47.6% and the three data sets presented high discrimination (Figure 1A). Then we performed a batch effect correction to minimize the total variance of component 1 and component 2, which decreased to 31.7% and the combined expression profile showed centralized distributions (Figure 1B), which indicated an effective correction. By utilizing the CERES algorithm with the data of glioma cell lines based on CRISPR/Cas9 screening, a total of 728 candidate genes were identified and 662 overlapped targets were determined in the expression profile (Supplementary Table S3). A univariate Cox proportional hazard regression screened for 265 significant genes for prognosis with *p* < 0.001 (Supplementary Table S4). To further find out the important genes in survival outcomes and reduce data dimension simultaneously, we employed a random survival forest, and 47 aggregated genes with a relative weight of more than 0.4 were selected for the analysis (Figure 2A, Supplementary Table S5). Then, NMF was performed and patients were divided into two clusters named C1 and C2 (Figures 2B,C, Supplementary Table S6).

### Characteristics of the Two Clusters

**FIGURE 3:**
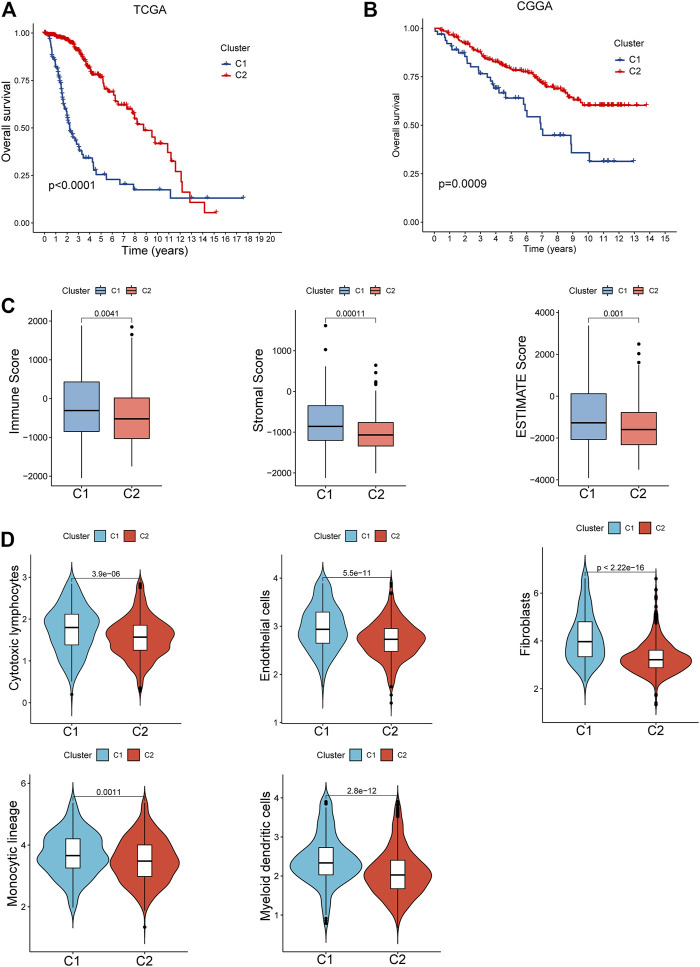
Clinical and cellular characteristics of the two clusters. **(A,B)** Survival differences between C1 and C2 of the TCGA and CGGA cohorts. **(C)** Comparison of the tumor purity between C1 and C2. **(D)** Tumor immune-infiltrating levels in the subtypes.

**FIGURE 4:**
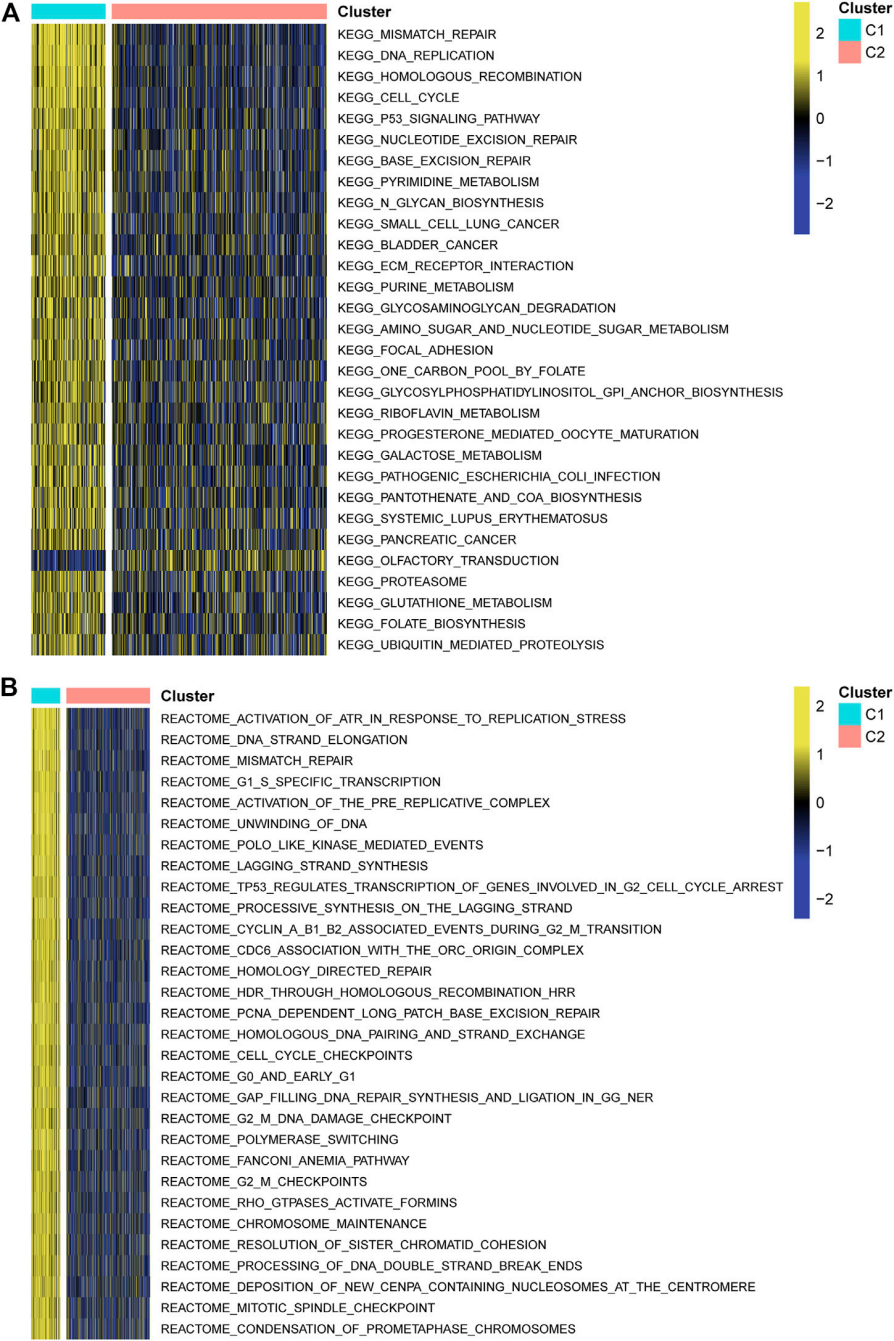
Functional enrichment analysis. **(A)** Kyoto Encyclopedia of Genes and Genomes. **(B)** Reactome pathways.

A survival analysis by log-rank test revealed that patients in C1 had worse survival compared to those from C2 of both TCGA (*p* < 0.0001, Figure 3A) and CGGA cohorts (*p* = 0.0009, Figure 3B). By calculating the ESTIMATE scores, immune score, and stromal score, it was implied that C1 exerted significantly higher immune (*p* = 0.004) and stromal components (*p* < 0.001) than C2, namely, lower tumor purity (*p* = 0.001, Figure 3C). Moreover, immune-filtrating levels estimated by microenvironment cell populations-counter (MCPcounter) suggested that C1 had higher infiltrating levels of cytotoxic lymphocytes (*p* < 0.001), endothelial cells (*p* < 0.001), fibroblasts (*p* < 0.001), monocytic lineage (*p* = 0.001), and myeloid dendritic cells (*p* < 0.001, Figure 3D). As to functional enrichment analysis, C1 presented activated Kyoto Encyclopedia of Genes and Genomes (KEGG) pathways such as the mismatch repair, cell cycle, homologous recombination, and p53 signaling pathway (Figure 4A). Reactome functions including DNA strand elongation, mismatch repair, activation of ATR in response to replication stress, and TP53 regulation of transcription of cell cycle genes were enriched in C1 (Figure 4B).

### Clinical Features of CRISPR/Cas9 Screening Potential Index Model Subtypes

**FIGURE 5:**
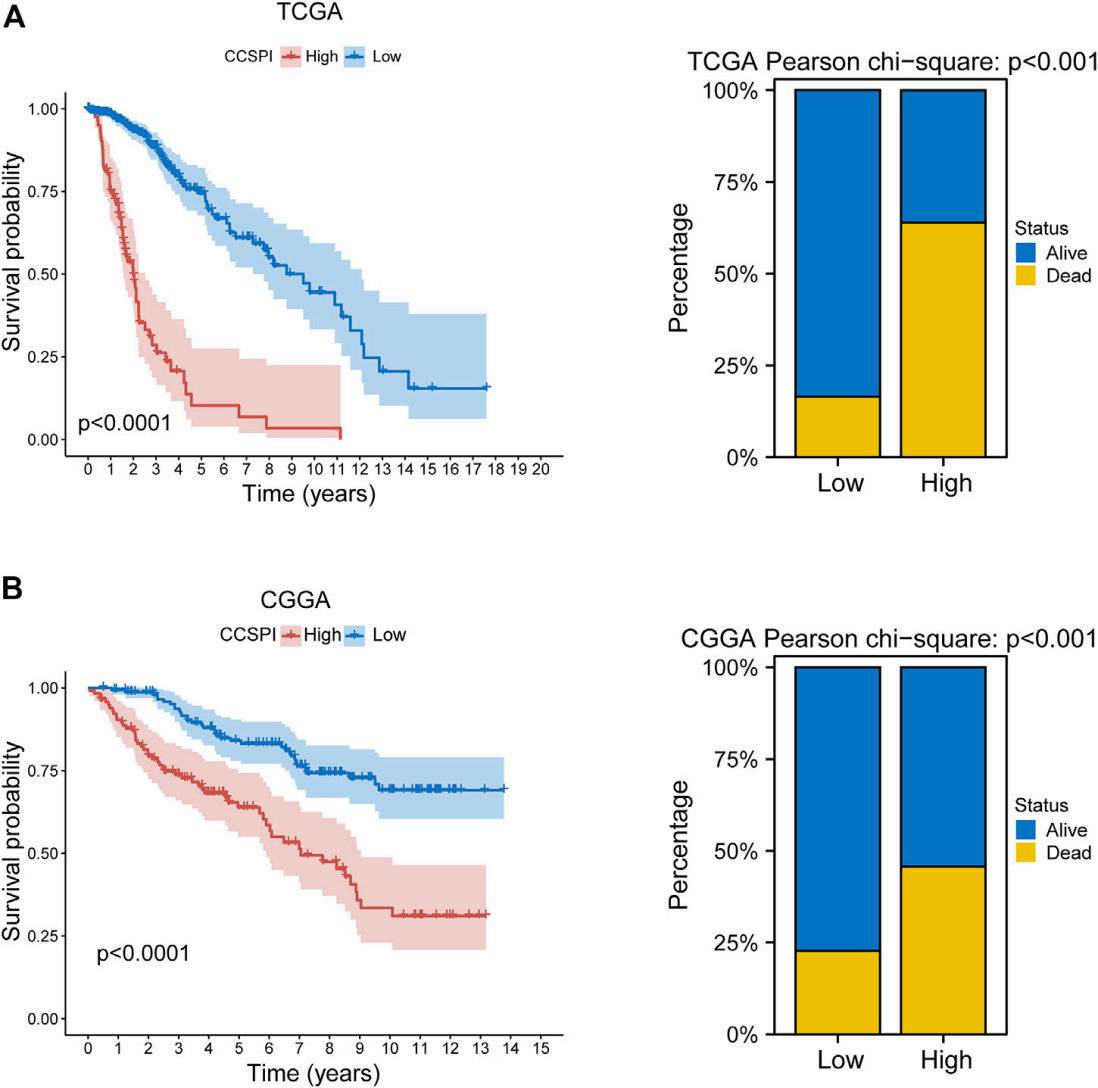
Survival outcomes of CCSPI stratifications. **(A)** Survival differences between low- and high-CCSPI groups of the training set and **(B)** validation set. (CCSPI: CRISPR/Cas9 screening potential index).

**FIGURE 6:**
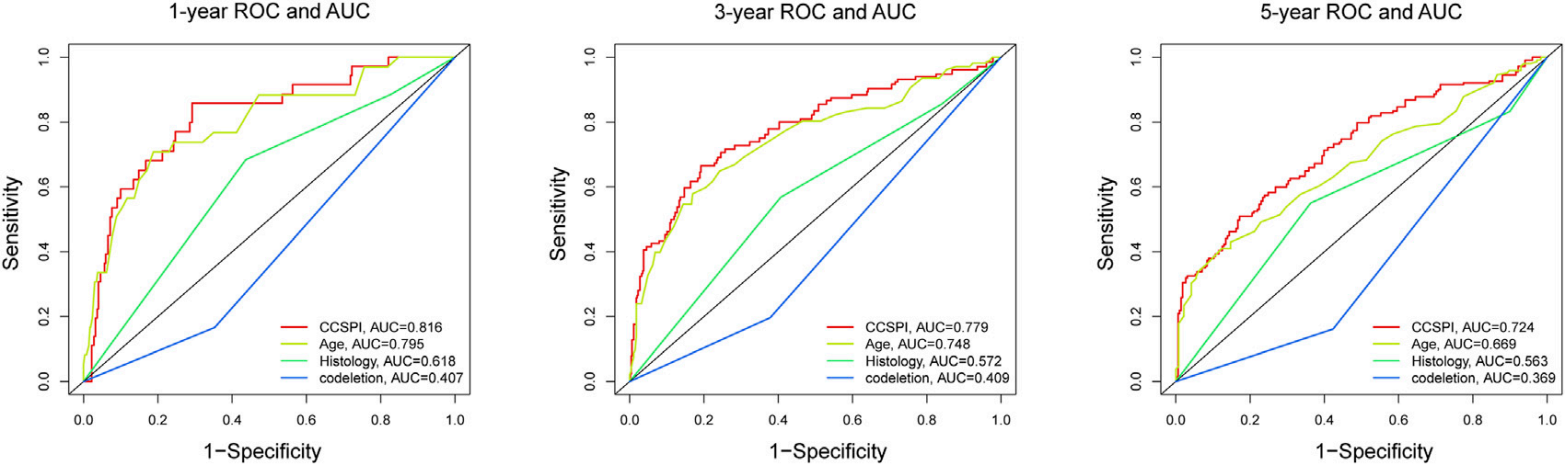
The 1-, 3-, and 5-year time-dependent receiver operating characteristic curves and area under the curve values evaluated the sensitivity and specificity of CCSPI, age, histology, and 1p/19q codeletion status.

**FIGURE 7:**
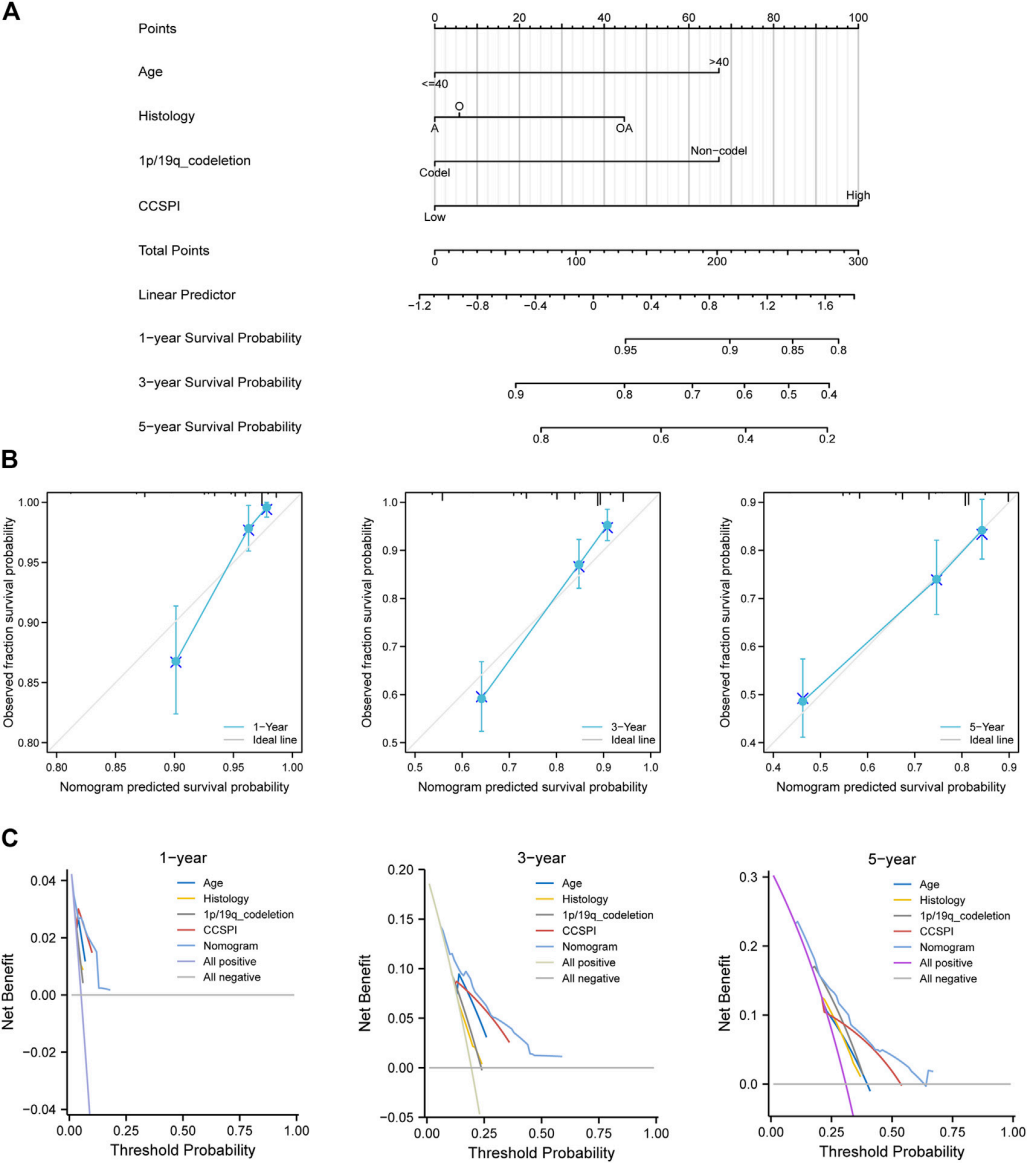
Development of combined diagnosis. **(A)** A nomogram model predicted the 1-, 3-, and 5-year survival probability of low-grade glioma patients. **(B)** Calibration curves of the nomogram. **(C)** Decision curve analysis. (A, astrocytoma; O, oligodendroglioma; OA, oligoastrocytoma).

There were two types of components to calculate CCSPI: a “risky component” including 40 genes and a “protective component” composed of seven genes (Supplementary Table S7). Patients were distinguished into low- and high-CCSPI groups at the cutoff of 0.4970 and 0.3807 in TCGA (n = 511) and CGGA (n = 291) datasets, respectively (Supplementary Table S8). Product-limit analysis and Pearson chi-squared test demonstrated a statistical difference in prognosis between the low- and high-CCSPI groups that patients with high CCSPI experienced poor survival in the training set and the validation set (both *p* < 0.001, Figures 5A,B). According to the results of survival analysis in distinctive clinicopathologic stratifications, it was illustrated that CCSPI subtypes had significant survival differences in clinicopathologic stratifications including age, gender, radiotherapy status, chemotherapy status, IDH mutation status, and 1p/19q codeletion status (Supplementary Figure S1). The 1-, 3-, and 5-year time-dependent ROCs and area under the curve values (AUCs) of CCSPI reached 0.816, 0.779, and 0.724, respectively, followed by age of 0.795, 0.748, and 0.669 (Figure 6). To verify the PH assumptions, we conducted several tests. The results of Schoenfeld’s test suggested that continuous data such as age and CCSPI and categorized data such as gender and IDH mutation status were not allowed to be enrolled in a Cox model (*p* < 0.05, Supplementary Figures S2A,B). Since the stratifications of age and CCSPI were included, which were confirmed by “log-minus-log” curves (Supplementary Figure S2C). Subsequently, a Cox proportional hazards model revealed that age (HR = 1.923, 95% CI = 1.435–2.577, *p* < 0.001), 1p/19q codeletion status (HR = 0.520, 95% CI = 0.322–0.840, *p* = 0.008), and CCSPI (HR = 2.649, 95% CI = 1.935–3.627, *p* < 0.001) could be independent factors for LGG prognostic prediction (Table 1). Moreover, the distributions of age (*p* < 0.001), histology (*p* < 0.001), IDH mutation (*p* < 0.001), and 1p/19q codeletion status (*p* < 0.001) were significantly different in the low- and high-CCSPI groups (Table 2). A nomogram was constructed with age, histology, 1p/19q codeletion status, and CCSPI (Figure 7A) with a C-index of 0.743 (95% CI = 0.725–0.760) showing promising predictive ability according to 1-, 3- and, 5-year calibration curves (Figure 7B) and DCA results (Figure 7C).

**TABLE 1:** Independent prognostic analysis by Cox analysis.

Variables	Total (N)	Univariate analysis		Multivariate analysis	
		Hazard ratio (95% CI)	*p* value	Hazard ratio (95% CI)	*p* value
Age	744	2.107 (1.584–2.803)	**<0.001**	1.923 (1.435–2.577)	**<0.001**
Histology	744				
Oligoastrocytoma	128	Reference			
Oligodendroglioma	281	0.607 (0.378–0.973)	**0.038**	0.684 (0.398–1.175)	0.169
Astrocytoma	335	1.184 (0.766–1.830)	0.447	0.647 (0.407–1.027)	0.065
1p/19q codeletion	744	0.389 (0.276–0.549)	**<0.001**	0.520 (0.322–0.840)	**0.008**
CCSPI	744	3.323 (2.518–4.386)	**<0.001**	2.649 (1.935–3.627)	**<0.001**

Bold values indicated statistical significance.

**TABLE 2:** Distributions of clinical features between the low- and high-CCSPI groups.

Variables		CCSPI		*p* value
		Low (N)	High (N)	
Age	≤40	312	70	**<0.001**
	>40	240	122	
Gender	Female	225	94	0.515
	Male	309	116	
Histology	Astrocytoma	194	141	**<0.001**
	Oligodendroglioma	243	38	
	Olioastrocytoma	115	13	
IDH mutation	Mutant	515	101	**<0.001**
	Wild	40	88	
1p/19q codeletion	Codel	236	20	**<0.001**
	Non-codel	316	172	

Bold values indicated statistical significance.

### Model Comparison

**FIGURE 8:**
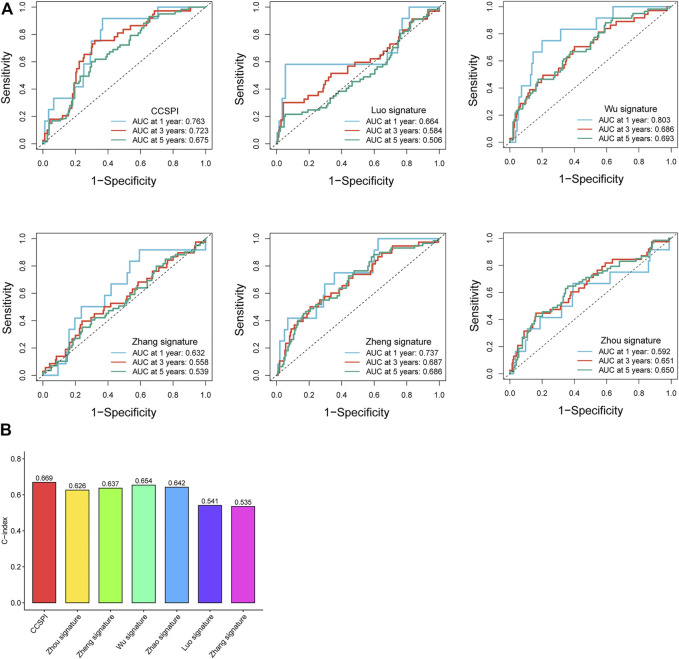
Comparison of different models. **(A)** 1-, 3- , and 5-year time-dependent receiver operating characteristic curves and area under the curve values. **(B)** C-indices of survival prediction based on distinct models.

According to the genes and corresponding coefficients for LGG prognostic prediction collected from several previous studies, we calculated the risk score and divided patients into low- and high-risk groups at the median cutoff. To evaluate the predicting ability, we performed a time-dependent ROC analysis, and it was noticeable that CCSPI exhibited the best performance with 1-, 3-, and 5-year AUCs of 0.763, 0.723, and 0.675, respectively, followed by Wu’s signature of 0.803, 0.686, and 0.693 and Zheng’s signature of 0.737, 0.687, and 0.686 (Figure 8A). Moreover, the C-index of CCSPI reached 0.669 followed by Wu’s signature of 0.654 and Zhao’s signature of 0.642 (Figure 8B).

### Biological Functions and Immune Correlations

**FIGURE 9:**
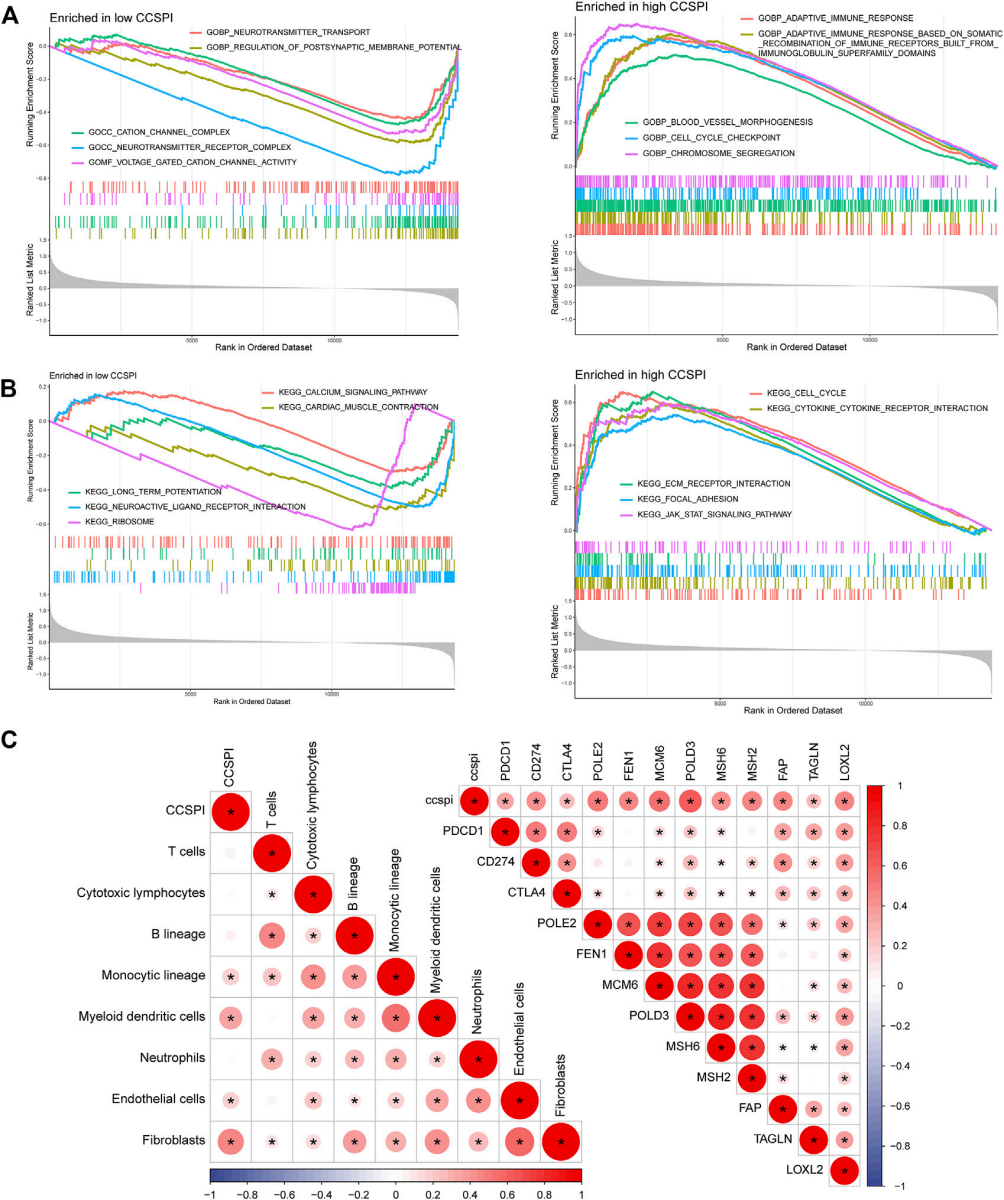
Functional enrichment and immune correlations with CCSPI. **(A)** Gene ontology terms in low- and high-CCSPI groups. **(B)** Kyoto Encyclopedia of Genes and Genomes pathways in low- and high-CCSPI groups. **(C)** Associations of CCSPI, immune checkpoint expressions, and tumor immune-infiltrating levels.

**FIGURE 10:**
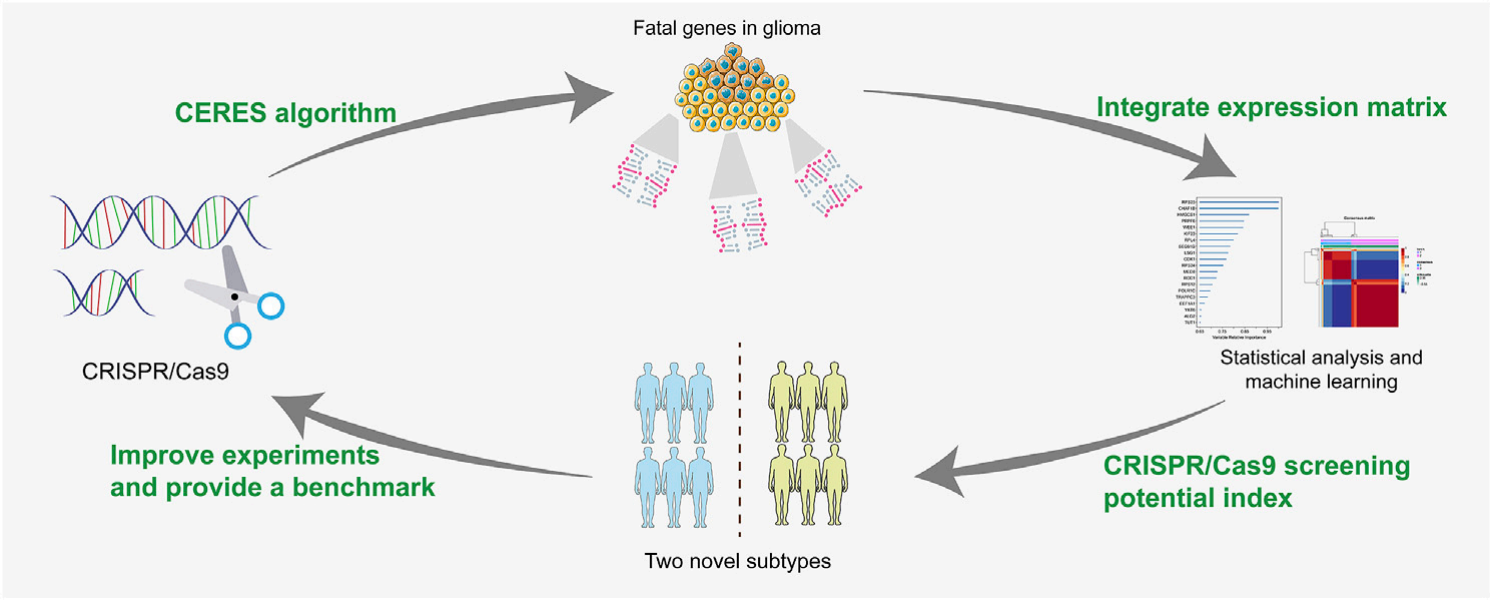
Workflow of this study.

Significant biological functions of gene ontology (GO) terms determined by gene set enrichment analysis (GSEA) showed the low-CCSPI group was enriched in the biological process including voltage-gated cation channel activity, neurotransmitter receptor complex, cation channel complex, regulation of postsynaptic membrane potential, and neurotransmitter transport, while high CCSPI presented chromosome segregation, cell cycle checkpoint, blood vessel morphogenesis, adaptive immune response based on somatic recombination of immune receptors built from immunoglobulin superfamily domains, and adaptive immune response (Figure 9A). Kyoto encyclopedia of genes and genomes (KEGG) pathways such as the ribosome, neuroactive ligand–receptor interaction, long-term potentiation, cardiac muscle contraction, and calcium signaling pathway were activated in the low-CCSPI group, while the Janus kinase-signal transducer and activator of transcription (JAK-STAT) signaling pathway, focal adhesion, ECM–receptor interaction, cytokine–cytokine receptor interaction, and cell cycle presented activation in the high-CCSPI group (Figure 9B). Moreover, we investigated the correlations between CCSPI, immune-infiltrating levels, and immune checkpoint expressions. Pearson correlation coefficients showed CCSPI was positively associated with immune cells such as fibroblasts, myeloid dendritic cells, monocytic lineage, and endothelial cells (Figure 9C). It also had significantly positive connections with conventional key targets such as PDCD1 (PD-1), CD274 (PD-L1), and CTLA-4 (Figure 9C). The diagram of this study was summarized in Figure 10.

## Discussion

CRISPR/Cas9 technology has been applied in diverse areas of cancer biology. But very few studies focus on bioinformatics with CRISPR/Cas9 in the prognosis and immunology of cancers. Previous research identified subgroups using lung adenocarcinoma cell lines (Ho et al., 2021) and determined breast cancer essential genes (Sun et al., 2021), which mainly analyzed the survival consequences. In the present study, we downloaded public datasets, including expression profiles and corresponding clinical phenotypes of glioma from several web servers. Using CRISPR/Cas9 filtering, candidates of glioma-lethal genes were selected for the analysis. We used a univariate Cox model and a random survival forest to screen for optimal prognostic genes. Then patients were categorized into two clusters (C1 and C2) by NMF. C1 presented worse prognosis and lower tumor purity, which is similar to previous findings that poor survival is partly caused by increasing fractions of neutrophils (Lin et al., 2021). This study revealed that C1 had more proportions of cytotoxic lymphocytes, endothelial cells, fibroblasts, monocytic lineage, myeloid dendritic cells, and lower purity, indicating different TME characteristics between C1 and C2. Since considerable alterations occur in the tumor microenvironment, which is tightly associated with immunotherapeutic efficiency (Codrici et al., 2022), varied treatments would be recommended for C1 and C2. For example, previous evidence showed that the microenvironment formed by the perivascular niche and composed of endothelial cells was correlated with tumor progression (do Nascimento et al., 2022) and activation of cytotoxic cells contributed to the mortality of cancer cells (Miyauchi and Tsirka, 2018). Hence regulating immune components or targeting stromal cells might be more applicable in C1.

In addition, we analyzed the biological features and clinical therapeutic effectiveness of C1 and C2. Pathways such as cell cycle, mismatch repair, p53 signaling pathway, and metabolism-related functions were activated in C1. The cell cycle closely connected with cell proliferation and apoptosis contributes to glioma management (Yin et al., 2021). P53 as a suppressor gene was previously verified to have a regulatory role in inducing apoptosis of glioma (Yang et al., 2022). A meta-analysis revealed that p53 expression was positively associated with a poor prognosis of glioma (Jin et al., 2016), agreeing with our findings. Metabolism not only plays a key part in target therapies but also in radiotherapy linked with DNA damage (Mudassar et al., 2020). It should be also pointed out that p53 controls a variety of metabolic processes such as the balance of glycolysis and oxidative features correlated to post-translational modification (Rahman et al., 2022). Thus, targeting the p53 signaling pathway and enhanced understanding of the mechanisms of the pathway regulating glioma development probably boost the survival rate of C1 patients. Another gene set of Reactome pathways illustrated that functions were all inhibited in C2 such as the diseases caused by the programmed cell death, activation of ATR in response to the replication stress, and TP53 regulation of transcription of cell cycle genes. These hallmarks were mostly correlated with programmed cell death signaling pathways, suggesting regulation of the cell cycle to promote apoptosis of cancer cells by changing the gene expressions such as knockdown of KIF11 (Liu et al., 2021) and SNRPG (Lan et al., 2020),which is conducive to cancer control for patients of C2 subtype.

To date, there are considerable risk models for LGG prognostic and immune prediction. Wang et al. (2020) developed a nomogram to predict LGG patients’ survival outcomes based on alternative splicing, which performed better than clinical markers such as age, grade, and IDH1 mutation status. Qu et al. (2020) and Qu et al. (2021a) constructed prognostic models for glioma using N6-methyladenine-related and autophagy-related genes, respectively. Although promising predicting efficacy was achieved, the accuracy of the models to evaluate the tumor microenvironment was not investigated. Moreover, the combination of LGG and GBM might lead to unreliability because the two subtypes of glioma differ a lot biologically. The computation of these models was all based on regression coefficients and gene expressions. The specific objective of this study was to create a novel index superior to the major clinicopathologic variables despite specific expression levels. Different from a previous study that used clinical markers to develop a scoring prognostic system (Qu et al., 2021b), the hallmark of this study was CRISPR/Cas9 screening enrolling a gene panel, which has more instructive information for further genome editing experiments. We defined a “risky component” and a “protective component” to develop a CCSPI by ssGSEA algorithm and classify the patients into low- and high-CCSPI groups. The high-CCSPI group exhibited a significantly worse overall survival than patients with low CCSPI. The predicting performances showed satisfactory sensitivity, accuracy, net benefits, and C-index, better than any other clinical features included in the current study, and the predictive efficacy of several existing models, suggesting that our model held great potential to predict prognosis.

To investigate the biological functions, we conducted a GSEA functional enrichment analysis of GO and KEGG. GO terms enriched in low-CCSPI included voltage-gated cation channel activity, neurotransmitter receptor complex, cation channel complex, regulation of postsynaptic membrane potential, and neurotransmitter transport. These are tightly related to neuromodulation. The topmost significantly enriched terms in the high-CCSPI contained immune response, anterior/posterior pattern specification, and blood vessel morphogenesis. It is noticeable that the immune response and anterior/posterior pattern specification are strongly related to glioma development, metastasis, and proliferation. KEGG analysis showed that the JAK-STAT signaling pathway, focal adhesion, ECM receptor interaction, cytokine-cytokine receptor interaction, and cell cycle were significant. Aberrant JAK-STAT signaling pathways in distinctive malignancies can cause misexpression of genes in additional carcinogenic pathways (Chang and Lai, 2019). Accordingly, regulating the JAK-STAT pathway targets may improve LGG diagnosis and treatment (Basheer et al., 2021; Li et al., 2021).

Furthermore, we analyzed the correlations of CCSPI, immune-infiltrating levels, and immune checkpoint expressions. CCSPI demonstrated relatively strong positive connections with fibroblasts, monocytic lineage, myeloid dendritic cells, and endothelial cells. Fibroblasts affect glioma pathogenesis and involve in glioma formation (Georgiou and Gkretsi, 2019). Hence a deeper understanding of the interplay between fibroblasts and glioma stem cell development using the CRISPR/Cas9 library perhaps brings innovative therapeutic approaches. CCSPI was also positively correlated with PD-1, PD-L1, POLE2, POLD3, LOXL2, etc. PD-1 and PD-L1 were extensively researched in glioma both clinically and biologically. It was recognized that PD-1 and PD-L1 are upregulated in glioma and patients with overexpressed PD-1/PD-L1 may benefit from the PD-1/PD-L1 checkpoint blockades such as nivolumab and temozolomide (Wang et al., 2019). Since we speculated that anti-PD1/PD-L1 therapies exhibit higher efficiency for high-CCSPI patients.

However, there were some shortcomings in the present study. The thesis only included pure computational analysis and did not engage with wet-lab strategies. External validation with more independent data cohorts and experiments such as immunohistochemistry, fluorescence staining, and CRISPR/Cas9 technology would be beneficial. We also failed to avoid information loss in the overlapping process. In future work, larger size of samples and more comprehensive approaches will be adopted, which is time consuming.

## Conclusion

In summary, we identified novel subtypes of LGG with relative lethal genes and constructed a CRISPR/Cas9 screening potential index to investigate LGG patients’ prognosis and immunologic features. The model will contribute to deepening our cognition of the crosstalk between the CNS and TME and providing more effective estimations of immunotherapeutic or chemotherapeutic benefits in different LGG subtypes.

## Data Availability

Data used in this study can be downloaded from TCGA (https://tcga-data.nci.nih.gov/tcga/), CGGA (http://www.cgga.org.cn/) and Depmap (https://depmap.org/portal/).

## Supplementary Material

Supplementary Figure S1 Survival differences between the low- and high-CCSPI groups of different clinical stratifications.

Supplementary Figure S2 Test of reliability of factors included in the Cox model. **(A)** Schoenfeld’s test of age and CCSPI. **(B)** Log minus log (LML) curves of gender, histology, IDH mutation status, and 1p/19q codeletion status test. **(C)** LML curves of age and CCSPI stratifications.

Supplementary Table S1 Details of glioma cell lines included in this study.

Supplementary Table S2 Strategies of risk score calculation from previously published studies.

Supplementary Table S3 Candidate glioma-lethal genes by CRISPR/Cas9 library screening.

Supplementary Table S4 265 significant genes in a univariate Cox regression.

Supplementary Table S5 Raw and relative importance of potentially prognostic candidate genes.

Supplementary Table S6 Clusters of LGG patients.

Supplementary Table S7 Risky and protective components.

Supplementary Table S8 CCSPI and corresponding stratifications at the optimal cutoff.

## Abbreviations

AUC, area under the curve; CCSPI: CRISPR/Cas9 screening potential index; CGGA, Chinese Glioma Genome Atlas; C-index, concordance index; CNS, central nervous system; CRISPR, clustered regularly interspaced short palindromic repeats; DCA, decision curve analysis; GDSC, Genomics of Drug Sensitivity in Cancer; GO, Gene Ontology; GSEA, gene set enrichment analysis; GSVA, gene set variation analysis; KEGG, Kyoto Encyclopedia of Genes and Genomes; KNN, K-Nearest Neighbor; LGG, low-grade glioma; MCPcounter, Microenvironment Cell Populations-counter; MSigDB, Molecular Signatures Database; PH, proportional hazard; ROC, receiver operating characteristic curve; TME, tumor microenvironment; TCGA, The Cancer Genome Atlas.
